# Supplementary material for: Self-management interventions for adults living with Chronic Obstructive Pulmonary Disease (COPD): The development of a Core Outcome Set for COMPAR-EU project
Source: PLoS One. 2021 Mar 1;16(3):e0247522. doi: 10.1371/journal.pone.0247522 (PMC7920347; doi:10.1371/journal.pone.0247522)
Supplement: S1 File — (PDF) [file pone.0247522.s001.pdf]

COPD

The patient perspective

To get a picture of what are the most important outcomes for patients living with chronic obstructive pulmonary disease (COPD), it is important to know what is the perspective of other patients around the world. For that purpose, we performed a scientific review of the published research studies.

We identified 27 reviews including more than 800 studies with thousands of patients living with COPD. The studies report patient's views, experiences, beliefs, and how they value in general the different outcomes related to the disease, and/or self-management interventions.

The results obtained are closely related with more than half of the outcomes that you have already assessed in the surveys that you have completed for the upcoming workshop in Berlin. The outcomes are organized in categories below.

Hope you find this information useful!

Self-management competences 1

Patient activation

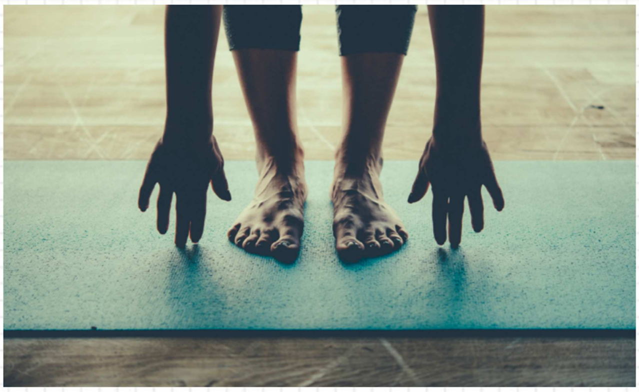

Patients describe that they adopted a active role in their own disease management, driven by their motivation to prevent another an attack of shortness of breath.

Knowledge

Patient's knowledge of COPD is reported as limited.
Patients with COPD refer not having enough information about aetiology, prognosis and treatment. They require further knowledge to guide their decision-making process to self-manage the disease.
Understanding COPD as a life-limiting condition is important to engage individuals in ongoing disease management and assisting individuals to interpret the emergence of symptoms as something more than just a normal part of life.

Participation and decision-making

COPD patients value as positive the experience of patient-centeredness care. This is especially true in rural area where patients have long-term relationship with health care providers.

Patients identify lack of discussion with health care professionals about lung health as a barrier for understanding.

Self-efficacy

Caring for the body through washing, dressing, cooking, and eating provide the bare minimum for survival and social acceptability, and loss or decline of any of these activities can be very traumatic.

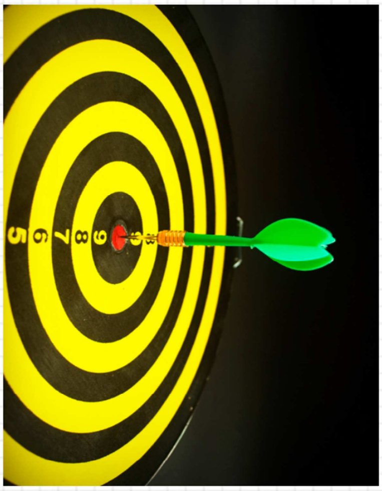

Health literacy

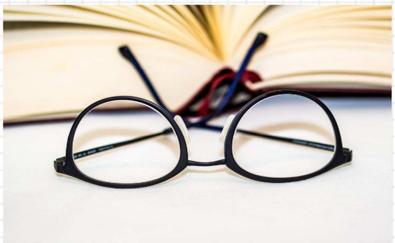

Some patients have difficulty to understand terminology, confuse COPD with asthma, do not understand the progressive and incurable nature of COPD and are confused regarding exercises and how to recognise and respond to exacerbations.

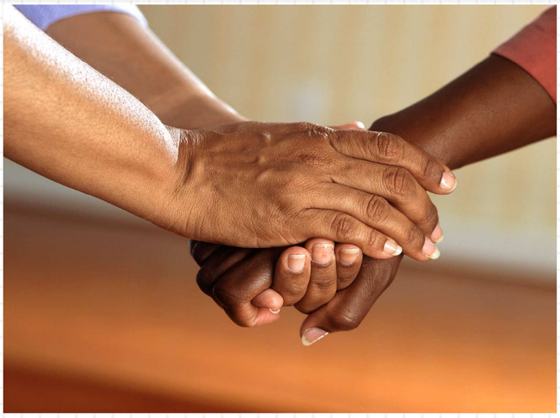

Self-management / Self-care behaviours

Taking medication or other treatments as advised (adherence)

In general, COPD patients adhere well to inhaler therapy and attend to pulmonary rehabilitation, because they fear dyspnoea and feel vulnerable.

Self-monitoring

Some patients become experts in what happens to their bodies. Based on previous experiences and constant observation of one's physical changes, they pay attention to the influence from outside, and the reactions to self-imposed adjustments or treatments.

Physical activity

Losing physical capabilities and having increased dependence increase patient's frailty, this fact causes distress since it undermines individual's feelings of self-worth and social identity.

Despite optimization of pharmacological treatments and interventions that directly target breathlessness and fatigue (such as pulmonary rehabilitation), some patients continue to experience high symptom burden and restriction in life activities.

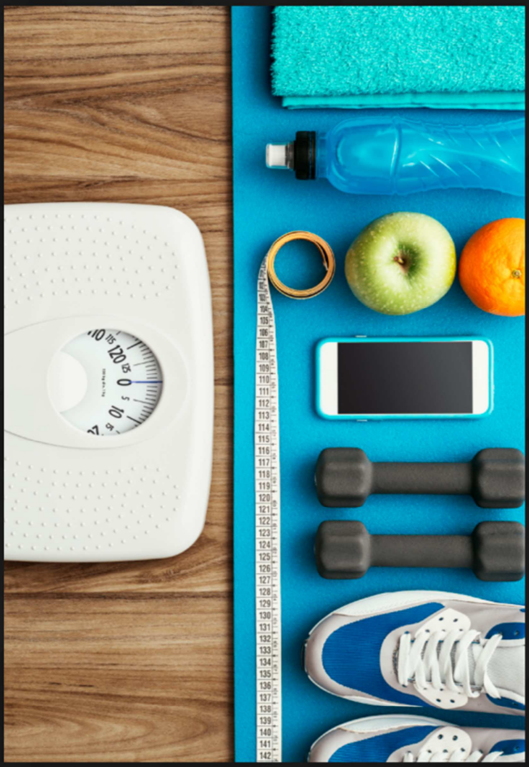

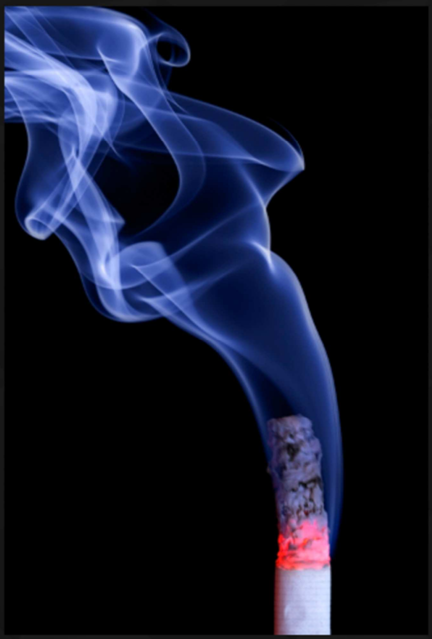

Smoking/ Smoking cessation

Smoking may impact negatively in patients since it may lead to feelings of guilt, self-blame and shame. These feelings, with the awareness that COPD is a progressive and incurable disease, may produce a sense of helplessness, which finally impacts on motivation for self-care, and adherence to treatment.

However, COPD smokers find the medical advice to quit conflicted with the increased desire to smoke as a coping strategy.

Disease management

3

Tiredness (fatigue)

The symptoms of COPD and the effects of the treatments disrupt the performance of activities of daily living of people with COPD, including their capacity to satisfy their physical and social needs.

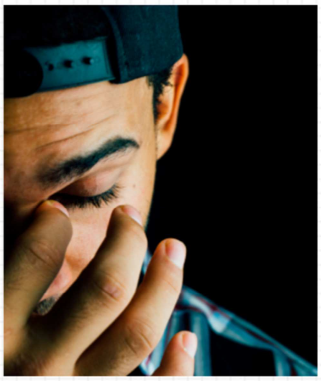

Breathlessness

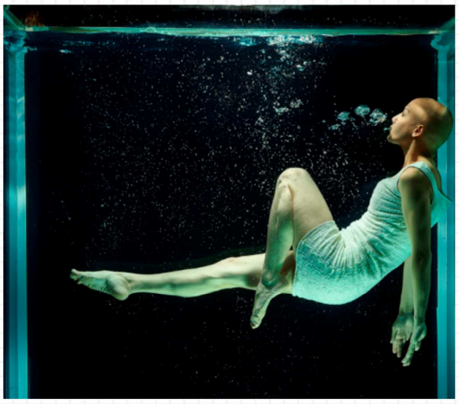

Breathlessness is one of the most prominent and threatening symptoms in the illness experience of COPD. It is sometimes experienced as painful, as a struggle and exhausting. Life is uncertain for the patient giving that COPD can unexpectedly get worse.

Exacerbation

Patients describe a COPD acute exacerbation as an over-whelming threat, being "near of death" or feeling the "shadow of death". Moreover some patients may experience a constant state of arousal and hyper vigilance to symptoms and body changes.

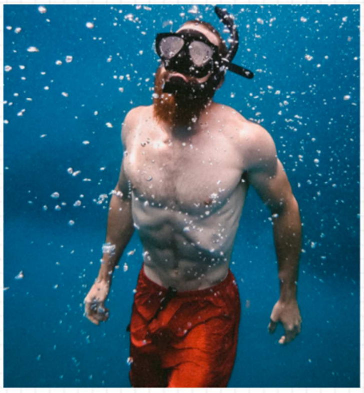

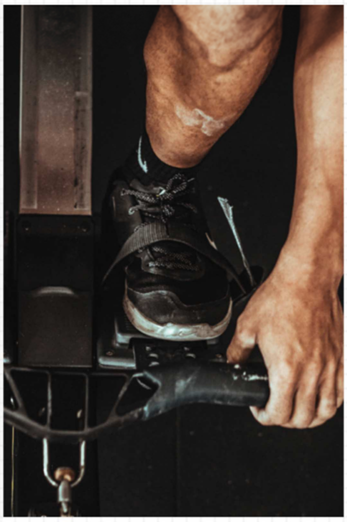

Muscle-strength /Exercise capacity

In Pulmonary Rehabilitation, patients describe as a facilitator the declining need for assistance for certain tasks once they have more confidence.

COPD patients distress because of their loss of physical capability, and increased dependence was evident across studies. This increased frailty also undermined individuals' feelings of self-worth and disrupted individuals' social identity.

Complications

4

Adverse events

Some COPD patients prefer to avoid adverse events more than other medication features, like timing of medication, cost, ease of use, and needing rescue treatment.
However, adverse events could be well tolerated, according to the extent of symptom's relief.

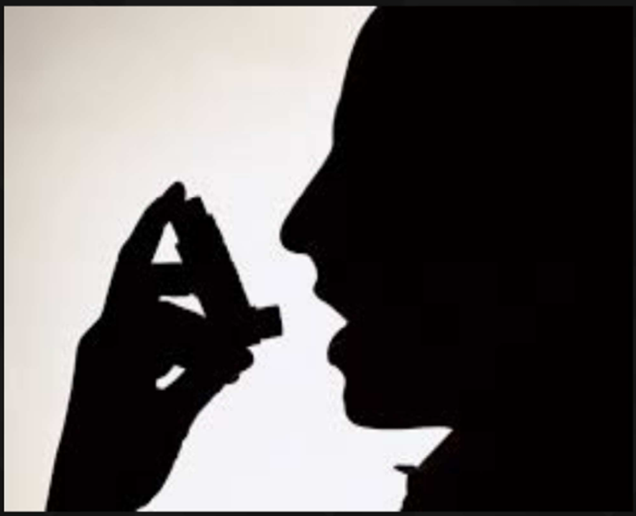

Evaluating COPD severity and complications with utilities

To assess what importance patients place on the different outcomes, researchers use the concept of "utility". Utilities measure how much value a person derives from something. In health, a utility describes a person's preference for living in a particular state of health- how much a person likes living in one health state (e.g. exacerbation).

Utilities are measured in a scale of 0 to 1, where 0 corresponds to death, and 1 corresponds to being in perfect health. All other health states fall between these extremes, and are described as a number greater than 0 and less than 1. Patients assign a value for each health state. Below we include some results from the scientific literature. Please be aware that estimates are averages, and that in general, patients report diverse results.

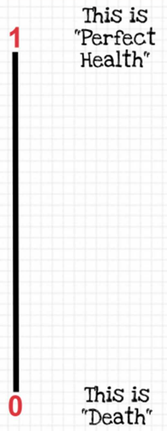

Health Utilities by COPD severity

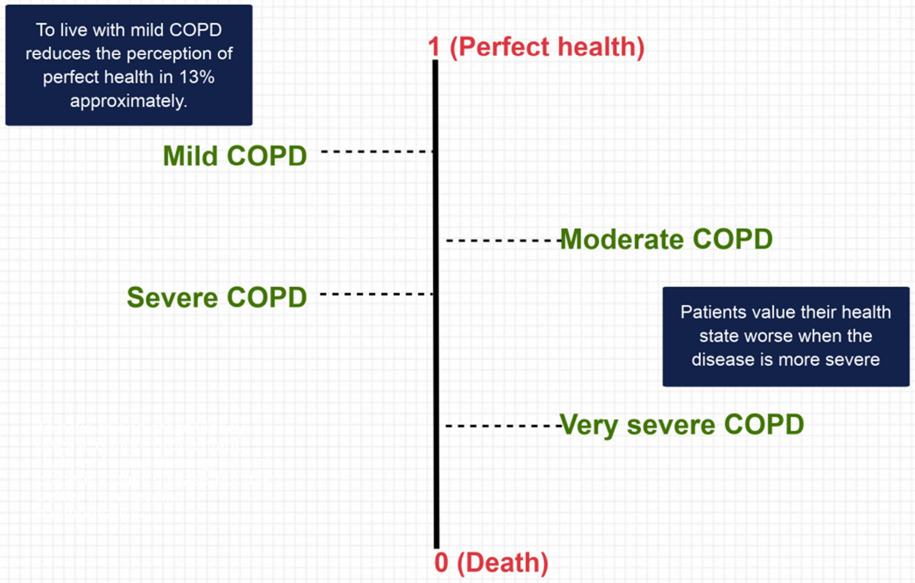

Health Utilities by breathlessness severity, and exacerbations

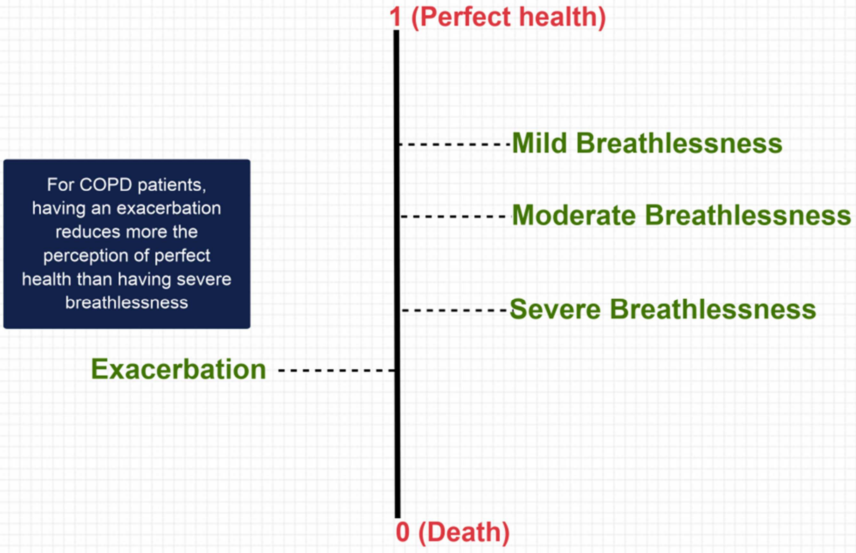

Other important outcomes

5

Symptom relief

In the literature we identified other outcomes that might be important for patients living with COPD. Extent of symptom relief is considered the most important outcome in two studies identified by a recent systematic review.

COPD patients prefer treatment focused on relieving pain and discomfort rather than extending life.

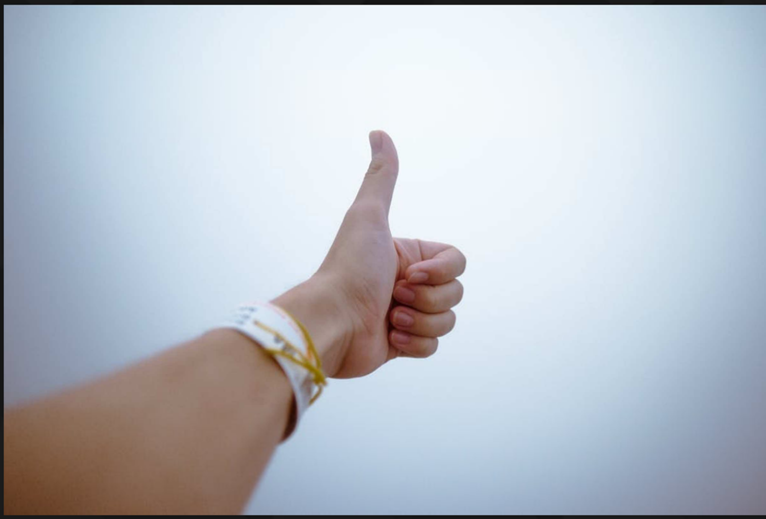

Physical functioning

6

Usual activities

Patients appear to be insightful about the effects of aging regarding the management of their condition, and seemed to be hopeful about their limitations, often expressing difficulties in separating disease and aging effects, and attributing symptoms as an inevitable consequence of disease.

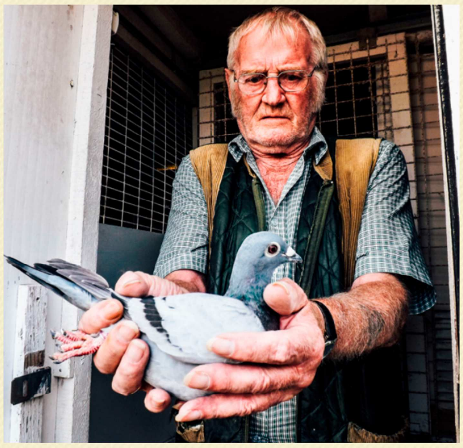

Work / normality

Patients experience loss of functional abilities such as working, social life, intimacy, liberty or future.

Patients feel isolation, and they often do not succeed in maintaining a normal image, getting frustrated, hurt and vulnerable.

Psychological functioning

7

Anxiety

Patients describe a dynamic relationship between dyspnoea and emotional functioning or anxiety, relating a “dyspnea-anxiety-dyspnea cycle”.

Depression

Anxiety, panic, and fear are commonly reported by patients, and associated with experiencing breathlessness, hospitalisation, as well as fearing a worsening of symptoms and death. This was something recognised by family/carers.

Hostility

Patient with COPD perceive stigma from their environment, this is significantly associated with dyspnoea and cough symptoms. Perceived stigma may be connected to poor treatment compliance in COPD.
Blame from others, family functioning were found to significantly correlate with self-blame.
COPD patients reported that higher perceptions of self-blame are associated with worse quality of life.

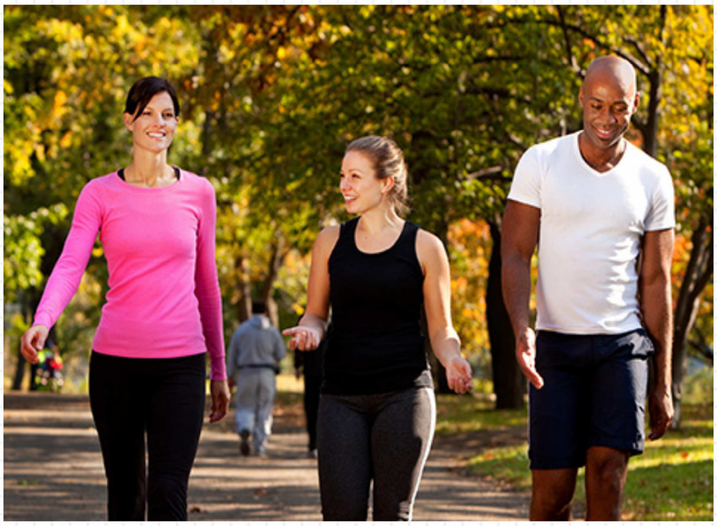

Stress

Illness perceptions that reflect attributing many symptoms to COPD, perceiving a low sense of control, and strong emotional perceptions (anxiety, depression, catastrophizing) might be associated with poor outcomes.

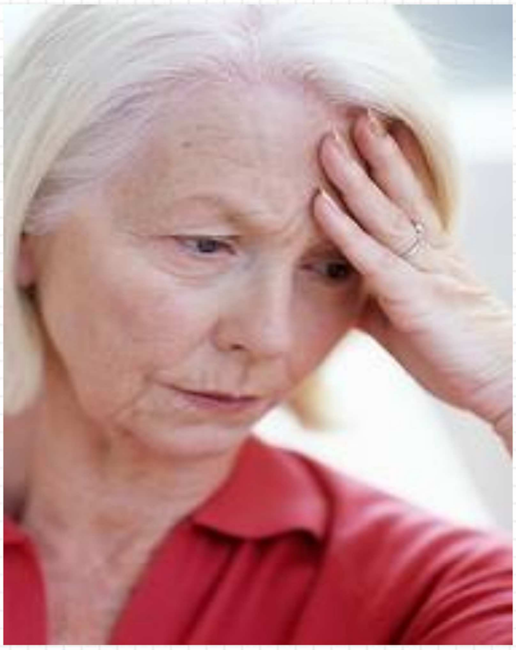

Self-esteem / Happiness / Coping

Self-reliance is a highly valued source of strength and personal control, especially for rural dwellers with chronic conditions; it helps to mitigate the experience of inadequate access to services.
The psychological effect of COPD could generate emotional needs that are not always easy to cope with.
The experience of living with COPD calls for self-reflection. Patients consider activities with others meaningful and satisfying. There are positive thoughts regarding deepened faith and spirituality.

Social relations and activities

8

Family Relationships / Friends

Connectedness is needed for leading a full life with COPD as it enables personal integrity over time. A loss of effectiveness is distressing, but it can be mitigated by help from a spouse, family member, or friend, enabled by and increasing connectedness.

The physical and psychological impact of COPD led individuals to live in an ever-shrinking and isolated world. Lost capability led to further disconnection and feelings of being a spectator.

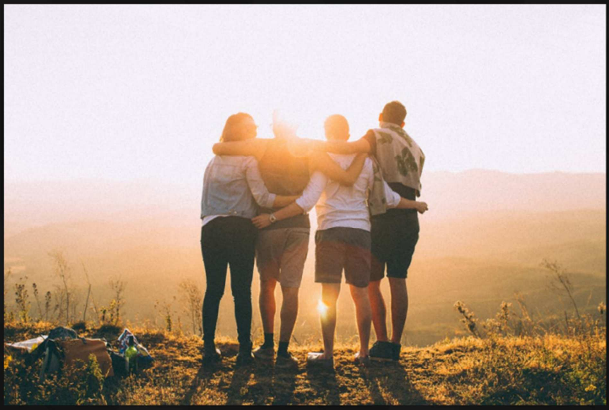

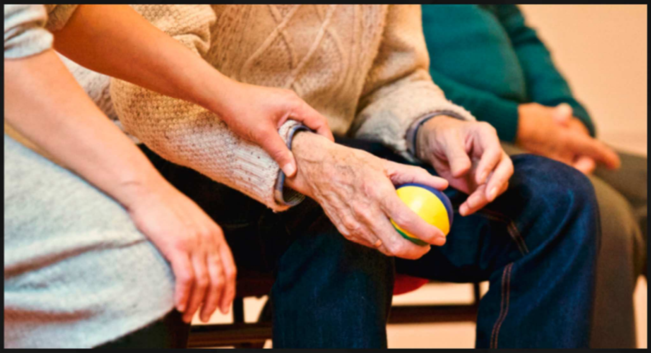

Social activities

COPD could impact negatively on social interactions due to reduced function and mobility, embarrassment from symptoms and fear of breathlessness.

Self-care may also be aimed at reducing the impact of COPD on social activities. COPD influences the social and family lives of those it affects, forcing them to renegotiate their family and social activities. These limitations required the use of personal and external resources to reduce the burden of the disease.

Caregivers quality of life & competences

9

Quality of life / Burden / Anxiety-Depression

Family members often experience a feeling of duty to care for their partners, which combined with a loss of intimacy could lead to psychological distress.

Family caregivers provided crucial support to patients with COPD, but they experienced considerable burdens themselves.

Carers' challenges often echo patients' challenges, and included anxiety, uncertainty about the future, helplessness, powerlessness, depression, difficulties maintaining employment, loss of mobility and freedoms, strained relationships, and growing social isolation.

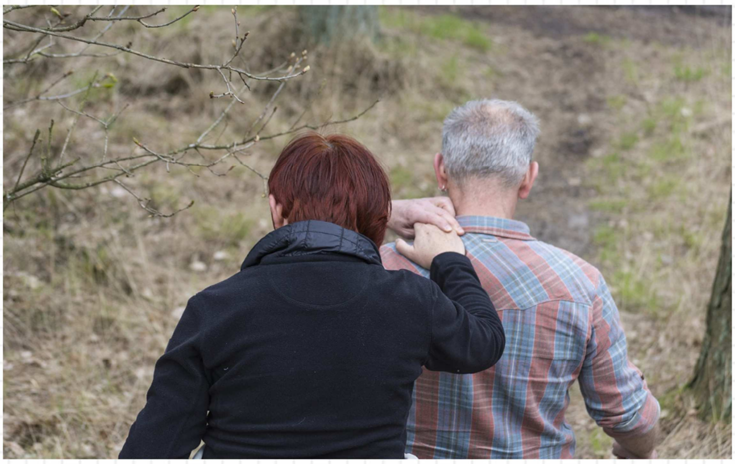

Satisfaction with care

Care satisfaction

Some patients see care received through telehealth to be as good as a visit from the nurse. These patients prefer to take their measurements themselves at home, as they feel comfortable there.

Other patients concern about the loss of personal contact with nurses, feeling that some services could not be delivered via telehealth, and finding face-to-face contact with healthcare professionals important.

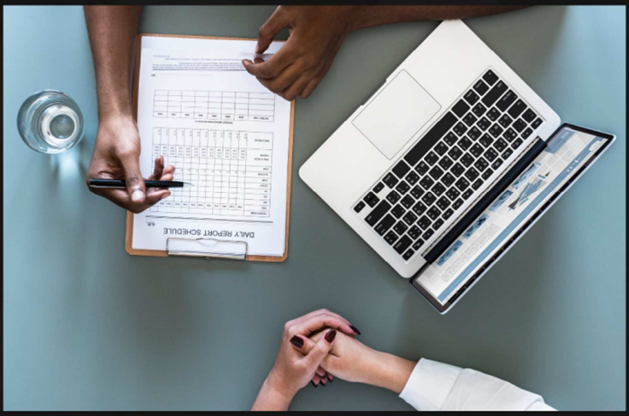

Patient-healthcare provider relation

Lack of discussion around lung health with health professionals is identified by patients as a one of the barriers for understanding.

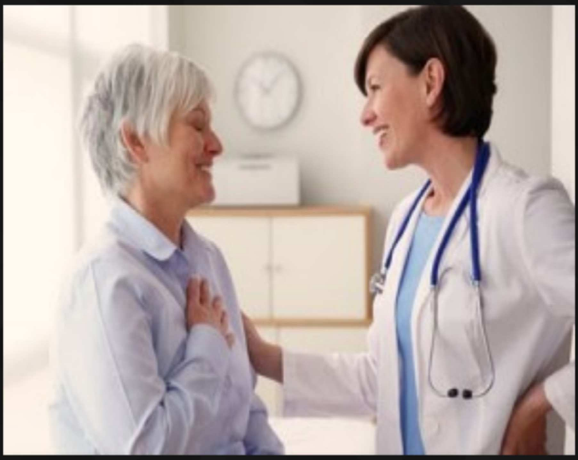

The patient feel s/he has enough information

Telehealth educates patient about their health, by providing more accurate information in smaller pieces over time. This helped to reinforce material, consequently giving patients a better understanding and awareness of their condition.

Visits or contact with healthcare providers

11

Primary care or outpatient visits / nurse / specialist

People with COPD refer having difficulty in accessing dedicated healthcare services. Furthermore, sanitary facilities without physical and architectural barriers are not always available, leading to further difficulty in accessing the care.
Patients typically seek initial treatment for an acute episode rather than for chronic early symptoms of COPD.

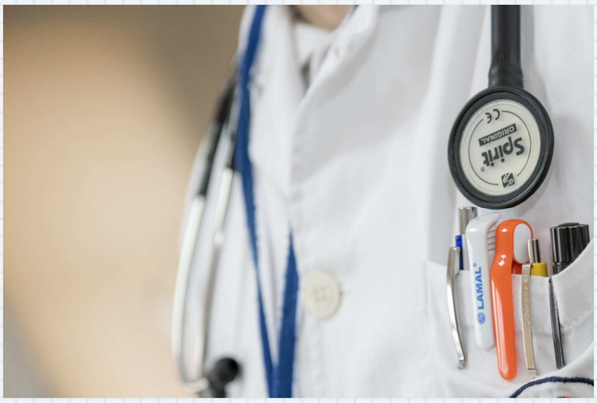

Hospitalisation

Lack of confidence in community-based services leads some patients to seek hospital admission, but patients also feel vulnerable when hospitalized. They may feel dependent on others for care or traumatized by hospital care routines. Upon hospital discharge following an exacerbation, patients may face new levels of uncertainty about their illness, prognosis, care providers, and supports.

Virtual

For some patients telehealth can be perceived as a way to increase dependency while for others it helps to enable self-care.

Patients articulate ambivalent attitudes to help-seeking, yet when experiencing intense breathlessness, help seeking is urgent, prompted by a fear of dying.

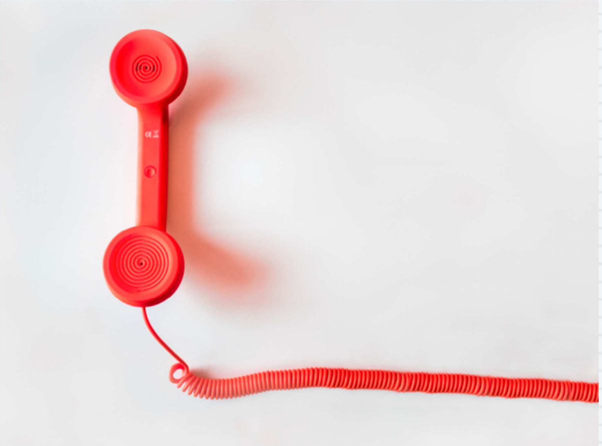

"This project has received funding from the European Union's Horizon 2020 research and innovation programmeunder grant agreement No 754936".

COMPAR-EU
