# Supplementary material for: Self-management interventions for adults living with Chronic Obstructive Pulmonary Disease (COPD): The development of a Core Outcome Set for COMPAR-EU project
Source: PLoS One. 2021 Mar 1;16(3):e0247522. doi: 10.1371/journal.pone.0247522 (PMC7920347; doi:10.1371/journal.pone.0247522)
Supplement: S3 File — (PDF) [file pone.0247522.s003.pdf]

Supplementary file 3. Consensus workshop participants characteristics – patients/patient representatives

ID	Age	Gender	Education	Occupation
2	45-54	Female	Master’s degree or equivalent	Patient advocate/patient representative affiliated with a patient organisation
5	> 65	Male	Master’s degree or equivalent	Individual patient affiliated with a patient organisation
6	> 65	Male	High school degree or equivalent	Individual patient affiliated with a patient organisation
7	45-54	Female	Master’s degree or equivalent	Patient advocate/patient representative affiliated with a patient organisation
9	> 65	Male	Master’s degree or equivalent	Patient advocate/patient representative affiliated with a patient organisation
