# Supplementary material for: Self-management interventions for adults living with Chronic Obstructive Pulmonary Disease (COPD): The development of a Core Outcome Set for COMPAR-EU project
Source: PLoS One. 2021 Mar 1;16(3):e0247522. doi: 10.1371/journal.pone.0247522 (PMC7920347; doi:10.1371/journal.pone.0247522)
Supplement: S4 File — (PDF) [file pone.0247522.s004.pdf]

Supplementary file 4. Consensus workshop participants characteristics – health professionals and researchers

ID	Gender	Country	Name of organization	Occupation
1	Male	Estonia	Department of Pulmonology, University of Tarfu	Professor
2	Male	Malta	Mater Dei Hospital / University of Malta	Consultant Respiratory Physician
3	Female	Ireland	Local Worlds	Individual patient affiliated with a patient organisation
4	Female	Ireland	Power’s Pharmacy, Kilmihil, Co Clare	Superintendent Community Pharmacist
5	Female	UK	Coventry University Hospital	Physician
