# Supplementary material for: Self-management interventions for adults living with Chronic Obstructive Pulmonary Disease (COPD): The development of a Core Outcome Set for COMPAR-EU project
Source: PLoS One. 2021 Mar 1;16(3):e0247522. doi: 10.1371/journal.pone.0247522 (PMC7920347; doi:10.1371/journal.pone.0247522)
Supplement: Attachment — Submitted filename: PONE-D-20-34041_reviewer.pdf [file pone.0247522.s005.pdf]

PLOS ONE

Self-management interventions for adults living with Chronic Obstructive Pulmonary Disease (COPD): The development of a Core Outcome Set for COMPAR-EU project.

--Manuscript Draft--

**Manuscript Number:**	PONE-D-20-34041
**Article Type:**	Research Article
**Full Title:**	Self-management interventions for adults living with Chronic Obstructive Pulmonary Disease (COPD): The development of a Core Outcome Set for COMPAR-EU project.
**Short Title:**	Core outcome set for self-management interventions in Chronic Obstructive Pulmonary Disease
**Corresponding Author:**	Ana Isabel González-González<br>Avedis Donabedian Research Institute<br>Barcelona, SPAIN
**Keywords:**	assessment, patient outcomes; patient centered care; chronic obstructive pulmonary disease; self management
**Abstract:**	Background<br>A large body of evidence suggests that self-management interventions (SMIs) may improve outcomes in chronic obstructive pulmonary disease (COPD). However, accurate comparisons of the relative effectiveness of SMIs are challenging, partly due to heterogeneity of outcomes across trials and uncertainty about relevance of these outcomes for patients. We aimed to develop a minimal core set of patient-relevant outcomes (COS) for SMIs trials in order to enhance comparability of interventions and ensure person-centred care.<br>Methods<br>We undertook an innovative approach consisting of four interlinked stages: i) Development of an initial catalogue of outcome domains from previous EU-funded projects/published studies, ii) Scoping review of reviews on patients and caregivers' perspectives for identifying outcomes of interest, iii) Two-round Delphi online survey with patients and patient representatives to rate the importance of outcomes identified, and iv) Face-to-face consensus workshop with patients, patient representatives, health professionals and researches for rating and selecting outcomes for the COS.<br>Results<br>From an initial list of 79 potential outcomes, 16 were included for the COS plus one supplementary outcome relevant to all participants. These were related to patient and caregiver knowledge/competence, self-efficacy, patient activation, self-monitoring, adherence, smoking cessation, COPD symptoms, physical activity, sleep quality, caregiver quality of life, activities of daily living, coping with the disease, participation and decision-making, emergency room visits/admissions and cost effectiveness.<br>Conclusion<br>The development of the COPD COS for the evaluation of SMIs will increase consistency in the measurement and reporting of outcomes across trials. In this way, it will also contribute to more personalized health care and more informed health decisions in clinical practice as patients' preferences regarding COPD outcomes are more systematically included.
**Order of Authors:**	Estela Camus-García
	Ana Isabel González-González
	Monique Heijmans
	Ena Niño
	Claudia Valli
	Jessica Beltran
	Hector Pardo-Hernández
	Lyudmil Ninov

Powered by Editorial Manager® and ProduXion Manager® from Aries Systems Corporation

	Valentina Strammiello
	Kaisa Immonen
	Dimitris Mavridis
	Marta Ballester
	Rosa Suñol
	Carola Orrego
**Additional Information:**	
**Question**	**Response**
**Financial Disclosure**<br><br>Enter a financial disclosure statement that describes the sources of funding for the work included in this submission. Review the submission guidelines for detailed requirements. View published research articles from *PLOS ONE* for specific examples.<br><br>This statement is required for submission and **will appear in the published article** if the submission is accepted. Please make sure it is accurate.<br><br>**Unfunded studies**<br>Enter: *The author(s) received no specific funding for this work.*<br><br>**Funded studies**<br>Enter a statement with the following details:<br>• Initials of the authors who received each award<br>• Grant numbers awarded to each author<br>• The full name of each funder<br>• URL of each funder website<br>• Did the sponsors or funders play any role in the study design, data collection and analysis, decision to publish, or preparation of the manuscript?<br>• **NO** - Include this sentence at the end of your statement: *The funders had no role in study design, data collection and analysis, decision to publish, or preparation of the manuscript.*<br>• **YES** - Specify the role(s) played.<br><br>* typeset	This work was supported by the EU Horizon 2020 research and innovation programme (grant agreement no. 754936).
**Competing Interests**<br><br>Use the instructions below to enter a	The authors have declared that no competing interests exist.

competing interest statement for this submission. On behalf of all authors, disclose any competing interests that could be perceived to bias this work—acknowledging all financial support and any other relevant financial or non-financial competing interests.<br><br>This statement **will appear in the published article** if the submission is accepted. Please make sure it is accurate. View published research articles from *PLOS ONE* for specific examples.<br><br>**NO authors have competing interests**<br><br>Enter: *The authors have declared that no competing interests exist.*<br><br>**Authors with competing interests**<br><br>Enter competing interest details beginning with this statement:<br><br>*I have read the journal's policy and the authors of this manuscript have the following competing interests: [insert competing interests here]*<br><br>* typeset	
**Ethics Statement**<br><br>Enter an ethics statement for this submission. This statement is required if the study involved:<br><br>• Human participants<br>• Human specimens or tissue<br>• Vertebrate animals or cephalopods<br>• Vertebrate embryos or tissues<br>• Field research<br><br>Write "N/A" if the submission does not require an ethics statement.<br><br>General guidance is provided below. Consult the submission guidelines for detailed instructions. **Make sure that all information entered here is included in the**	Ethical approval was obtained by the Clinical Research Ethics Committee of the coordinator partner, Avedis Donabedian Research Institute, in March 2018.

**Methods section of the manuscript.**<br><br>**Format for specific study types**<br><br>**Human Subject Research (involving human participants and/or tissue)**<br>• Give the name of the institutional review board or ethics committee that approved the study<br>• Include the approval number and/or a statement indicating approval of this research<br>• Indicate the form of consent obtained (written/oral) or the reason that consent was not obtained (e.g. the data were analyzed anonymously)<br><br>**Animal Research (involving vertebrate animals, embryos or tissues)**<br>• Provide the name of the Institutional Animal Care and Use Committee (IACUC) or other relevant ethics board that reviewed the study protocol, and indicate whether they approved this research or granted a formal waiver of ethical approval<br>• Include an approval number if one was obtained<br>• If the study involved *non-human primates*, add *additional details* about animal welfare and steps taken to ameliorate suffering<br>• If anesthesia, euthanasia, or any kind of animal sacrifice is part of the study, include briefly which substances and/or methods were applied<br><br>**Field Research**<br><br>Include the following details if this study involves the collection of plant, animal, or other materials from a natural setting:<br>• Field permit number<br>• Name of the institution or relevant body that granted permission	
**Data Availability**<br><br>Authors are required to make all data underlying the findings described fully available, without restriction, and from the time of publication. PLOS allows rare exceptions to address legal and ethical concerns. See the PLOS Data Policy and FAQ for detailed information.	Yes - all data are fully available without restriction

Powered by Editorial Manager® and ProduXion Manager® from Aries Systems Corporation

A Data Availability Statement describing where the data can be found is required at submission. Your answers to this question constitute the Data Availability Statement and **will be published in the article**, if accepted.<br><br>**Important:** Stating 'data available on request from the author' is not sufficient. If your data are only available upon request, select 'No' for the first question and explain your exceptional situation in the text box.<br><br>Do the authors confirm that all data underlying the findings described in their manuscript are fully available without restriction?	
**Describe where the data may be found in full sentences. If you are copying our sample text, replace any instances of XXX with the appropriate details.**<br><br>• If the data are **held or will be held in a public repository**, include URLs, accession numbers or DOIs. If this information will only be available after acceptance, indicate this by ticking the box below. For example: *All XXX files are available from the XXX database (accession number(s) XXX, XXX.*).<br>• If the data are all contained **within the manuscript and/or Supporting Information files**, enter the following: *All relevant data are within the manuscript and its Supporting Information files.*<br>• If neither of these applies but you are able to provide **details of access elsewhere**, with or without limitations, please do so. For example:<br><br>*Data cannot be shared publicly because of [XXX]. Data are available from the XXX Institutional Data Access / Ethics Committee (contact via XXX) for researchers who meet the criteria for access to confidential data.*<br><br>*The data underlying the results*	All relevant data are within the manuscript and its Supporting Information files.

presented in the study are available from (include the name of the third party and contact information or URL).<br>• This text is appropriate if the data are owned by a third party and authors do not have permission to share the data.<br><br>* typeset	
Additional data availability information:	

Powered by Editorial Manager® and ProduXion Manager® from Aries Systems Corporation

Manuscript

Click here to access/download;Manuscript;COPD_COS_Manuscript_20110

TITLE

Self-management interventions for adults living with Chronic Obstructive Pulmonary Disease (COPD): The development of a Core Outcome Set for COMPAR-EU project.

AUTHORS

Estela Camus-García

Avedis Donabedian Research Institute (FAD), Universitat Autonòma de Barcelona, Barcelona, SPAIN

Email: estela.camus@gmail.com

Ana Isabel González-González

Avedis Donabedian Research Institute (FAD), Universitat Autònoma de Barcelona, Barcelona, SPAIN

Red de Investigación en Servicios de Salud en Enfermedades Crónicas (REDISSEC), SPAIN

Email: aigonzalez@fadq.org

Monique Heijmans

Netherlands Institute for Health Services Research (NIVEL), Utrecht, THE NETHERLANDS

Email: m.heijmans@nivel.nl

Ena Niño de Guzmán

Iberoamerican Cochrane Centre Barcelona, Department of Clinical Epidemiology and Public Health, Biomedical Research Institute San Pau (IIB Sant Pau), Barcelona, SPAIN
Email: enino@santpau.cat

Claudia Valli
Iberoamerican Cochrane Centre Barcelona, Biomedical Research Institute San Pau (IIB Sant Pau), Barcelona, SPAIN
Department of Paediatrics, Obstetrics, Gynaecology and Preventive Medicine, Universidad Atónoma de Barcelona, Barcelona, SPAIN.
Email: claudia.valli89@gmail.com

Jessica Beltran
Iberoamerican Cochrane Centre Barcelona, Biomedical Research Institute San Pau (IIB Sant Pau), Barcelona, SPAIN
Email: jbeltranp@santpau.cat

Hector Pardo-Hernández
Iberoamerican Cochrane Centre Barcelona, Biomedical Research Institute San Pau (IIB Sant Pau) - CIBER Epidemiología y Salud Pública (CIBERESP), Barcelona, SPAIN
Email: hectorpardohernandez@gmail.com

Lyudmil Ninov
European Patients' Forum (EPF)
Email: lyudmil.ninov@eu-patient.eu

Valentina Strammiello
European Patients' Forum (EPF)
Email: valentina.strammiello@eu-patient.eu

Kaisa Immonen
European Patients' Forum (EPF)
Email: kaisa.immonen.charalambous@eu-patient.eu

Dimitris Mavridis
Department of Primary Education, University of Ioannina, Ioannina, GREECE.
Email: dmavridi@uoi.gr

Marta Ballester
Avedis Donabedian Research Institute (FAD), Universitat Autònoma de Barcelona, Barcelona, SPAIN.
Red de Investigación en Servicios de Salud en Enfermedades Crónicas (REDISSEC), SPAIN.
Email: mballester@fadq.org

Rosa Suñol
Avedis Donabedian Research Institute (FAD), Universitat Autònoma de Barcelona, Barcelona, SPAIN.

Red de Investigación en Servicios de Salud en Enfermedades Crónicas (REDISSEC), SPAIN.
Email: rsunol@fadq.org

Carola Orrego
Avedis Donabedian Research Institute (FAD), Universitat Autònoma de Barcelona, Barcelona, SPAIN.
Red de Investigación en Servicios de Salud en Enfermedades Crónicas (REDISSEC), SPAIN.
Email: corrego@fadq.org

CORRESPONDING AUTHOR

Ana Isabel González-González
Avedis Donabedian Research Institute (FAD), Universitat Autònoma de Barcelona, Calle Provenza 293 pral, 08037 Barcelona, SPAIN
Email: aigonzalez@fadq.org

ABSTRACT

Background

A large body of evidence suggests that self-management interventions (SMIs) may improve outcomes in chronic obstructive pulmonary disease (COPD). However, accurate comparisons of the relative effectiveness of SMIs are challenging, partly due to heterogeneity of outcomes across trials and uncertainty about the importance of these outcomes for patients. We aimed to develop a core set of patient-relevant outcomes (COS) for SMIs trials to enhance comparability of interventions and ensure person-centred care.

Methods

We undertook an innovative approach consisting of four interlinked stages: i) Development of an initial catalogue of outcomes from previous EU-funded projects and/or published studies, ii) Scoping review of reviews on patients and caregivers' perspectives to identify outcomes of interest, iii) Two-round Delphi online survey with patients and patient representatives to rate the importance of outcomes, and iv) Face-to-face consensus workshop with patients, patient representatives, health professionals and researches to develop the COS.

Results

From an initial list of 79 potential outcomes, 16 were included in the COS plus one supplementary outcome relevant to all participants. These were related to patient and caregiver knowledge/competence, self-efficacy, patient activation, self-monitoring, adherence, smoking cessation, COPD symptoms, physical activity, sleep quality, caregiver quality of life, activities of daily living, coping with the disease, participation and decision-making, emergency room visits/admissions and cost effectiveness.

Conclusion

The development of the COPD COS for the evaluation of SMIs will increase consistency in the measurement and reporting of outcomes across trials. It will also contribute to more personalized health care and more informed health decisions in clinical practice as patients' preferences regarding COPD outcomes are more systematically included.

INTRODUCTION

Chronic Obstructive Pulmonary Disease (COPD) is one of the major causes of morbidity and mortality worldwide (1,2). The economic and social burden related to COPD are expected to increase over the coming decades due to the continued exposure to COPD risk factors and the increasing aging of the world's population (3). COPD prevalence varies across countries and across different groups within countries (i.e., being male, older and former or current smoker) (4). It is directly related to the prevalence of tobacco smoking, although in many countries outdoor and indoor air pollution constitute major risk factors (5,6).

The literature suggests that self-management interventions (SMIs) may improve clinical outcomes, quality of life and reduce costs of chronic conditions, including COPD (7,8). A Cochrane systematic review showed that SMIs along with support from health professionals improve health-related quality of life while decreasing hospitalizations and emergency department visits of COPD patients (9).

Two recent studies, the COMET (10) and the PIC-COPD (11) showed the potential of SMIs for reducing exacerbations and mortality in integrated case management, as well as for increasing physical activity. However, synthesizing the evidence on the relative effectiveness of SMIs for COPD is challenging due to heterogeneity of interventions, lack of clear definitions of self-management components, and variability in the outcomes reported. Moreover, systematic reviews on SMI effectiveness have found insufficient data for some outcomes, which may be suggestive of selective reporting (9,12,13).

SMIs can only be compared across studies when they share some common outcomes. In addition, it is important to create consensus about what outcomes are especially

relevant to assess the effects of SMI and how they should be measured. By reaching consensus of a standardized set of outcomes that should be minimally measured and reported in future COPD clinical trials, we will ensure the comparativeness of results and synthesis of the evidence across studies (14). This outcome set should be relevant for all stakeholders, but especially for patients, as they are the ones primarily responsible for the daily management of their disease. In this study we propose a systematic approach to develop a Core Outcome Set [COS] for measuring the effectiveness of SMIs interventions in COPD from the perspective of both patients and health care professionals. This study is part of COMPAR-EU, an EU-funded project designed to bridge the gap between current knowledge and practice on SMIs in four chronic conditions one of them being COPD.

MATERIAL AND METHODS

The COS for SMIs in COPD patients was developed in accordance with the Core Outcome Measures for Effectiveness Trials (COMET) Handbook (14) and the Core Outcome Set-STAndards for Development (COS-STAD) guidelines (15). This study was conducted according to a protocol previously published (16). The COMPAR-EU COS approach involved four interlinked stages that are described below and summarized in **Figure 1.**

[Insert here: Figure 1 on Overall study design of Core Outcome Set for COPD].

Stage 1. Development of an initial catalogue of outcomes

Data sources and searches

We developed an initial catalogue of outcomes from a literature review of previous EU [illegible]ded projects that included overviews of systematic reviews focusing on SMIs for chronic diseases: i) PRO-STEP (Promoting Self-Management for Chronic Diseases in the

EU) (17) and ii) EMPATHiE (Empowering Patients in the Management of Chronic Diseases) (18). We additionally searched for COPD COS in relevant organizations databases such as COMET (10) and ICHOM [International Consortium for Outcomes Health Measurement] (19).

Study selection

We included systematic reviews and individual studies that reported outcomes on SMIs for patients with COPD. We excluded systematic reviews that did not report a final list of outcomes or individual studies where the final list of outcomes was not developed considering patients' input, experiences or values and preferences. We screened title and abstracts and assessed eligible full-text articles independently. In case of disagreement, reviewers reached consensus or consulted with a referee. Reviewers checked references from included studies to identify other potentially eligible studies.

Data extraction

Pairs of authors independently extracted the following data from eligible studies: i) study database, ii) type of publication (i.e., published COS, literature review or systematic review), iii) age groups, and iv) list of outcomes.

Data synthesis

We tabulated and classified the identified outcomes into the following seven categories following the process for the development of the COMPAR-EU taxonomy (20): i) empowerment components, ii) adherence to expected self-management behaviours, iii) clinical outcomes, iv) patient and informal caregivers' quality of life, v) perceptions and/or satisfaction with care, vi) healthcare use and vii) costs. The research team reviewed and discussed outcomes and merged them when possible.

Through an iterative process, an external clinician and researcher reviewed and discussed the resulting list of outcomes with multidisciplinary experts from the COMPAR-EU consortium. We prepared a definition of each outcome with the participation of all COMPAR-EU team members. Experts in health literacy and patient representatives adapted the resulting list of outcomes and presented it in plain language. This list of outcomes was to be used in the first round of the Delphi process (Stage 3).

Stage 2. Scoping review of reviews on perspectives of patients and their caregivers regarding self-management

We conducted a scoping review of reviews (21) to identify and describe key concepts related to outcomes by exploring patients' and caregivers' preferences and experiences when coping with COPD and its self-management.

Data sources and searches

We searched MEDLINE, CINAHL and PsycINFO from inception until February 2018. We applied a content search strategy for values and preferences (22) in combination with terms specific for COPD. We used review filters available in each database.

Study selection

We included reviews of quantitative, qualitative or mixed-methods studies that explored the perspectives, experiences, values and preferences of patients and caregivers on SMIs for COPD.

Data extraction

In a previously pilot-tested data extraction form, we collected the general characteristics and main findings of each review.

Data synthesis

We conducted a descriptive thematic synthesis including the identification of codes, descriptive themes and main themes relevant to outcomes of SMI for COPD. We paired main emerging themes with the subdomains of the COMPAR-EU taxonomy (20) and mapped the correspondence between themes and the initial catalogue of outcomes. We developed infographics illustrating themes to be used as aid materials during the consensus workshop.

Stage 3. Delphi survey (Round I and II)

To prioritize the outcomes identified, we administered two-round modified Delphi online surveys to a convenience sample. Our sample included patients and patients' representatives to ensure that we address outcomes that matter to patients as well as to other stakeholders.

Study population and eligibility criteria

We included a[illegible]s diagnosed with COPD and patients' representatives who were able to understand and speak English and provided informed consent to participate through the web platform hosting the Delphi rounds.

Recruitment strategy

Participants were identified within the European Patient Forum's EU wide membership network of more than 70 patient organizations (23) and other patient groups (e.g., those involved in ICHOM) (19). Recruitment started and concluded in February 2018 and ended in May 2018.

Delphi survey

The first and second Delphi rounds took place between May 2018 and June 2018. All participants received an online survey with the outcomes and definitions. They also received weekly reminders and were able to return to the questionnaire within a 3-

week period. Some of the participants were supported by their local organizations when completing it. Participants were asked "*How important do you think the following outcomes are to measure the success of self-management in people with COPD?*". COPD outcomes for SMIs were prioritized during the two-round Delphi process using a 1 to 9 Likert scale (1 being the least and 9 being the most important for the self-management of COPD).

During the second round, participants were able to see ratings (average score) from the first round and thus, adjust, confirm or rethink their answers. This process enabled participants to rate the most relevant SMIs outcomes for COPD according to their perspective

Data synthesis and analysis

All outcomes were categorized into three groups based on the level of agreement of ratings from the two-round Delphi online surveys as follows **(Table 1)**: i) Group 1 or "high consensus and high importance outcomes", ii) Group 2 or "low consensus and mixed importance outcomes" and iii) Group 3 or "high consensus of moderate and low importance outcomes".

[Insert here: Table 1 on Categories of outcomes by level of agreement].

Stage 4. Consensus workshop and final COPD COS

The final stage of the COPD COS development was a two and a half-day, in-person consensus workshop held in July 2018 in Berlin (Germany). The aim of the workshop was achieving consensus on the most important outcomes to include in the final COPD COS for the COMPAR-EU project. COPD patients and patients' representatives who participated in the two Delphi rounds, health professionals and researchers were invited to participate.

Participants received the results of the two-round Delphi survey (stage 3), and infographics illustrating themes, by outcome, from the scoping review (stage 2) one week before the consensus workshop which were used as additional material for free consultation. We organised outcomes according to a preliminary version of the outcome COMPAR-EU taxonomy (20). We sorted them by level of agreement as described previously. The COMPAR-EU research team led step-by-step the flow of the discussion to address potential discrepancies across stakeholders **(Figure 2)**. The group worked on prioritizing and selecting a maximum of 15 outcomes and up to five supplementary outcomes from those that had remained from the Delphi survey results. Participants selected outcomes through an iterative voting (secret vote) and discussion process. Outcomes that were closely related were merged. Once a preliminary list was agreed upon after voting and discussing, participants further reviewed the included outcomes and reached an agreement on the final version of the COPD COS.

[Insert here: Figure 2 on Consensus workshop decision diagram].

RESULTS

Stage 1. Development of an initial catalogue of outcomes

Study selection

The literature review of previous EU funded projects (PRO-STEP(17) and EMPATHiE (18)) identified records focusing on SMIs in chronic diseases in general. We included 22 systematic reviews specific to COPD (24,25,34–43,26,44,45,27–33) from PRO-STEP. The additional search in COMET (10), ICHOM (19) and through snowballing yielded 23

articles. After full-text appraisal, we included five studies (46–50); one study was excluded because it did not report the list of outcomes (51).

Study characteristics

The five included studies reported: i) a summary of outcomes for COPD pharmacological trials from lung function to biomarkers created by the American Thoracic Society / European Respiratory Society Task Force (46), ii) a review of instruments used to measure symptom response in pharmacological trials (47), iii) a review of articles determining themes identified as most important by COPD patients for any aspect of care of COPD (48), iv) a review assessing clinical outcomes in COPD mainly used on current published data (50), and v) a study addressing patient preferences regarding the expectations related to treatment of COPD.

List of outcomes and outcomes classification

We identified 79 outcomes for the initial list of outcomes. We classified outcomes into seven predefined subdomains based on a taxonomy for SMIs (20). **Table 2** presents the outcomes classification.

[Insert here: Table 2 on List of COPD outcomes and classification].

Stage 2. Scoping review of reviews on perspectives of patients and their caregivers regarding self-management

Study selection

Among the 1,031 unique screened references, 27 reviews were included comprising more than 800 studies.

Study characteristics

Of the 27 reviews for COPD, 16 (59%) were qualitative evidence synthesis (52,53,62–67,54–61), six (22%) quantitative systematic reviews (48,68–72), four (15%) were mixed methods research synthesis (73–76), and one (4%) was a literature review (77). The number of included studies ranged from five (70) to 213 (72). The majority of the reviews (n=22, 82%) included only the patients' perspective. The phenomena of interest addressed among reviews were preferences on health states of COPD (n=5, 18%), experiences with the process of self-management (n=14, 52%) and experiences with self-management interventions (n=8, 30%).

Main themes related to SMI outcomes for COPD

We identified 21 main themes, which are presented in **Table 3**. These themes were classified under i) empowerment components, ii) adherence to the expected self-management behaviours, iii) clinical-related outcomes, iv) quality of life of patients and caregivers, v) perceptions and/or satisfaction with care, vi) health care use, vii) costs. **Table 3** presents the subdomains of the COMPAR-EU taxonomy and the related identified themes for COPD.

[Insert here: Table 3 on Main themes related to COPD outcomes according to the subdomains from the COMPAR-EU taxonomy].

Mapping of themes

Of the 79 outcomes from the initial catalogue of outcomes, 45 were covered in the thematic synthesis (57%). All outcomes of the subdomain "empowerment components" were informed by the scoping review findings (n=4, 100%), while the subdomain "costs" was the least informed subdomain (n=1, 20%). **Figure 3** reports the number of outcomes informed by the thematic synthesis of the scoping review.

[Insert here: Figure 3 on Mapping of themes per COMPAR-EU taxonomy subdomain].

COPD infographic

An infographic was developed for the final consensus workshop including the main findings and topic related images (Stage 4). The infographic included the outcomes of the initial catalogue informed by the scoping review, classified according to the preliminary version of the outcome taxonomy (**Supplementary file 1**). This material and results from the Delphi rounds were sent to the consensus workshop participants (stage 4) one week in advance.

[Insert here link to Supplementary file 1 on Infographic COPD].

Stage 3. Two-round modified Delphi survey

Participants were invited via email. Nine participants accepted the invitation to participate and completed round I and round II of the Delphi online survey. Of these, five (56%) were patients and four (44%) were patient advocates or patients' representatives. Six (67%) were men, five (56%) were over 65 years old and seven (78%) had higher education (master or doctoral equivalent) (**Supplementary file 2**).

[Insert here link to Supplementary file 2 on Delphi online survey participants characteristics].

After the two-round Delphi survey, 23 (29%) of the 79 included outcomes were voted as high agreement on high importance (Group A: ≥70% of participants voted 7 to 9 on the Likert scale), eight (10%) as high agreement on non-importance (Group C: ≥70% of participants voted 1 to 3 on the Likert scale) and 48 (61%) voted intermediate agreement on importance (Group B) (**Table 1**).

Stage 4. Consensus workshop and final COPD core outcome set

Five of the nine patients or patients' representatives that participated in the Delphi online survey and five health professionals and researchers participated in the face-to-face consensus meeting. Five members of the COMPAR-EU research team participated as facilitators (**Supplementary files 3 and 4**).

[Insert here link to Supplementary file 3 on Consensus workshop participants characteristics patients/patients' representatives].

[Insert here link to Supplementary file 4 on Consensus workshop participants characteristics –health professionals and researchers].

The consensus workshop resulted in 16 outcomes for COPD plus 1 supplementary outcome (**Table 4**). Within these 16 outcomes, Delphi participants rated eight (50%) as high agreement on high importance, seven (44%) as low agreement and mixed importance rating, and one (6%) as high consensus of moderate and low support. The forme[illegible]oping with the disease, including depression and anxiety, was included in the final version of the COS (as par[illegible] the quality of life category and psychological functioning subcategory. Knowledge was part of the high consensus and high importance outcomes and was rendered as a supplementary outcome.

[Insert here: Table 4 on COMPAR-EU COS for COPD].

DISCUSSION

Main findings

The final COS for COPD included 16 outcomes plus 1 supplementary outcome. It represents the first COS developed based on patient preferences for evaluating SMIs in adults living with COPD. The COS incorporated results from a literature review complemented by a participatory process involving patients and patient

representatives along with health professionals and researches in all stages of the process.

Our results in the context of previous research

To the best of our knowledge, this is the first COS where a significant part of the work was led by patient representatives' organizations (EPF). Although various approaches have been described to develop COS (78,79), it is still uncertain which are the most appropriate. We chose to follow an iterative mixed-method approach involving different methodologies used in previous studies (80). The COS we present is novel since it focuses specifically on SMIs for COPD. Previous studies have focused on COPD management or other conditions (81–83). Spargo et al. (81) developed a COS for trials investigating the long-term management of bronchiectasis combining an overview of systematic reviews and qualitative studies and a Delphi panel that included mostly health professionals who rated the importance of each outcome initially selected. Verburg et al. (82) developed a standard set of outcome domains and proposed measures for patients with COPD for Dutch primary care physical therapy using a consensus-driven modified RAND-UCLA appropriateness method with relevant stakeholders. Jones et al. (83) created a priority list of measures for a combined COPD and heart failure exercise rehabilitation program through a stakeholders consensus event.

Strengths and limitations

The first list of COS was mainly based on the results from a literature review on three comprehensive overviews of systematic reviews performed in a previous project (PRO-STEP). As such, it incorporates a robust body of evidence vested in previous projects. The COS development aligns with current methodological guidelines for COS

development, as it included a participatory process of patients, patient representatives and other key stakeholders in all stages of the process (14). Therefore, the resulting COS is strongly based on patient preferences while also incorporating the viewpoints of health professionals, researchers and patients' representatives.

Outcome definitions were adapted to user-friendly language by EPF, which has extensive experience working with and presenting research material to patients in an intelligible manner. This ensured the comprehensibility of the process and the applicability of the results.

Our work is subject to some limitations. The number of participants during the Delphi process was small. We are confident that this shortcoming was overcome via the further deliberations that took place during the workshop. For the workshop, since only five of nine patients from Delphi participated in the consensus, we cannot rule out potential of attrition bias. Lastly, our sample in the Delphi and the consensus workshop may not be entirely representative of the population of patients with COPD. They could represent very motivated individuals or well-informed patients with high education or digital skills. However, and given the resources available, it would not have been feasible to adopt methodology different from electronic surveys (e.g., in-person interviews or surveys) to reach out to participants that are more diverse.

Implications for practice and research

The identified COS will inform a series of systematic reviews and network meta-analysis (NMA) about the effectiveness of SMIs as part of the COMPAR-EU project. We are confident that the COPD COS reflects the preferences of all key stakeholders and that it might be applicable with context adaptation to wide range of settings across Europe and the world. Future research evaluating SMIs for COPD should, as a

minimum, include the outcomes in the proposed COS. Further work is needed to identify and provide guidance on the most appropriate measures for each outcome.

CONCLUSIONS

We have developed the first COS for SMIs in COPD. This COS will increase consistency in the reporting of results that are relevant to patients across trials evaluating SMIs for COPD. This COS will enhance evidence synthesis of COPD patient-relevant outcomes and will decisively support research and overall field development. It will improve informed health-decision making in clinical practice and will increase the certainty of evidence to guide policy-making and clinical practice regarding SMI in COPD patients.

ACKNOWLEDGMENTS

The authors would like to thank all patients and patient representatives who participated in this project. Their real life-experiential knowledge was invaluable for the development of this COS.

The COMPAR-EU group: Aretj-Angeliki Veroniki (University of Ioannina, Department of Primary Education), Carlos Canelo-Aybar (Iberoamerican Cochrane Centre – Biomedical 400 Research Institute Sant Pau (IIB Sant Pau)), Christos Christogiannis (University of Ioannina, Department of Primary Education), Claudio Alfonso Rocha Calderón (Iberoamerican Cochrane Centre – Biomedical Research Institute Sant Pau (IIB Sant Pau) and CIBER de Epidemiología y Salud Pública (CIBERESP)), Cordula Wagner (Netherlands Institute for Health Services Research, (NIVEL)), Giorgos Seitidis (University of Ioannina, Department of Primary Education), Jany Rademakers (Netherlands Institute for Health Services Research, (NIVEL)), Katerina-Maria Kontouli

(University of Ioannina, Department of Primary Education), Karla Salas (Iberoamerican Cochrane Centre – Biomedical Research Institute Sant Pau (IIB Sant Pau)), Kevin Pacheco-Barrios (Avedis Donabedian Research Institute (FAD)), Kostas Aligiannis (European Patients' Forum), Marieke van der Gaag (Netherlands Institute for Health Services Research, (NIVEL)), Matthijs Michaël Versteegh (Institute for Medical Technology Assessment, Erasmus University Rotterdam, Rotterdam), Montserrat León (Iberoamerican Cochrane Centre – Biomedical Research Institute Sant Pau (IIB Sant Pau)), Nina Adrion (OptiMedis AG), Oliver Groene (OptiMedis AG), Pablo Alonso (Iberoamerican Cochrane Centre – Biomedical Research Institute Sant Pau (IIB Sant Pau) and CIBER de Epidemiología y Salud Pública (CIBERESP)), Rune Poortvliet (Netherlands Institute for Health Services Research, (NIVEL)), Sofia Tsokani (University of Ioannina, Department of Primary Education), Stavros Nikolakopoulos (University of Ioannina, Department of Primary Education), Stella Zevgiti (University of Ioannina, Department of Primary Education), Jessica Zafra (Avedis Donabedian Research Institute (FAD)).

CONTRIBUTIONS

EC and AIG wrote the initial draft of the manuscript. CO is the guarantor of the project. All authors participated in the developed of the core outcome sets activities. They also read, provided input and approved the final manuscript.

FUNDING

This work was supported by the EU Horizon 2020 research and innovation programme (grant agreement no. 754936).

ETHICS STATEMENT

Ethical approval was obtained by the Clinical Research Ethics Committee of the coordinator partner, Avedis Donabedian Research Institute, in March 2018.

PATIENTS AND PUBLIC INVOLVEMENT

Patients were a key component of this phase of the COMPAR-EU project. Their interests are represented by the European Patient Forum (EPF). Ethical approval was obtained by the Clinical Research Ethics Committee of University Institute for Primary Care Research – IDIAP Jordi Gol. All patients and other stakeholders provided written informed consent prior to participation.

REFERENCES

1. Lozano R, Naghavi M, Foreman K, Lim S, Shibuya K, Aboyans V, et al. Global and regional mortality from 235 causes of death for 20 age groups in 1990 and 2010: a systematic analysis for the Global Burden of Disease Study 2010. Lancet. 2012 Dec;380(9859):2095–128.
2. Vos T, Flaxman AD, Naghavi M, Lozano R, Michaud C, Ezzati M, et al. Years lived with disability (YLDs) for 1160 sequelae of 289 diseases and injuries 1990–2010: a systematic analysis for the Global Burden of Disease Study 2010. Lancet. 2012 Dec;380(9859):2163–96.
3. Mathers CD, Loncar D. Projections of Global Mortality and Burden of Disease from 2002 to 2030. Samet J, editor. PLoS Med. 2006 Nov 28;3(11):e442.

4. Whitmore G, Aaron S, Gershon A, Gao Y, Yang J. Influence of country-level differences on COPD prevalence. Int J Chron Obstruct Pulmon Dis. 2016 Sep;Volume 11:2305–13.

5. Eisner MD, Anthonisen N, Coultas D, Kuenzli N, Perez-Padilla R, Postma D, et al. An Official American Thoracic Society Public Policy Statement: Novel Risk Factors and the Global Burden of Chronic Obstructive Pulmonary Disease. Am J Respir Crit Care Med. 2010 Sep;182(5):693–718.

6. Salvi SS, Barnes PJ. Chronic obstructive pulmonary disease in non-smokers. Lancet. 2009 Aug;374(9691):733–43.

7. Newham J, Presseau J, Heslop-Marshall K, Russell S, Ogunbayo O, Netts P, et al. Features of self-management interventions for people with COPD associated with improved health-related quality of life and reduced emergency department visits: a systematic review and meta-analysis. Int J Chron Obstruct Pulmon Dis. 2017 Jun;Volume 12:1705–20.

8. Murphy LA, Harrington P, Taylor SJ, Teljeur C, Smith SM, Pinnock H, et al. Clinical-effectiveness of self-management interventions in chronic obstructive pulmonary disease: An overview of reviews. Chron Respir Dis. 2017 Aug 24;14(3):276–88.

9. Lenferink A, Brusse-Keizer M, van der Valk PD, Frith PA, Zwerink M, Monninkhof EM, et al. Self-management interventions including action plans for exacerbations versus usual care in patients with chronic obstructive pulmonary disease. Cochrane Database Syst Rev. 2017 Aug 4;

10. Kessler R, Casan-Clara P, Koehler D, Tognella S, Viejo JL, Dal Negro RW, et al. COMET: a multicomponent home-based disease-management programme

versus routine care in severe COPD. Eur Respir J. 2018 Jan 11;51(1):1701612.

11. Rose L, Istanboulian L, Carriere L, Thomas A, Lee H-B, Rezaie S, et al. Program of Integrated Care for Patients with Chronic Obstructive Pulmonary Disease and Multiple Comorbidities (PIC COPD +): a randomised controlled trial. Eur Respir J. 2018 Jan 11;51(1):1701567.

12. Shaw G, Whelan ME, Armitage LC, Roberts N, Farmer AJ. Are COPD self-management mobile applications effective? A systematic review and meta-analysis. npj Prim Care Respir Med. 2020 Dec 1;30(1):11.

13. Kaptein A, Fischer M, Scharloo M. Self-management in patients with COPD: theoretical context, content, outcomes, and integration into clinical care. Int J Chron Obstruct Pulmon Dis. 2014 Sep;907.

14. Williamson PR, Altman DG, Bagley H, Barnes KL, Blazeby JM, Brookes ST, et al. The COMET Handbook: version 1.0. Trials. 2017 Jun 20;18(S3):280.

15. Kirkham JJ, Davis K, Altman DG, Blazeby JM, Clarke M, Tunis S, et al. Core Outcome Set-STAndards for Development: The COS-STAD recommendations. PLOS Med. 2017 Nov 16;14(11):e1002447.

16. Ballester M, Orrego C, Heijmans M, Alonso-Coello P, Versteegh MM, Mavridis D, et al. Comparing the effectiveness and cost-effectiveness of self-management interventions in four high-priority chronic conditions in Europe (COMPAR-EU): a research protocol. BMJ Open. 2020 Jan 19;10(1):e034680.

17. PRO-STEP Consortium. Promoting self-management for chronic diseases in the EU-PROSTEP project. 2018.

18. EMPATHiE Consortium. EMPATHiE, empowering patients in the management of chronic diseases. Final Summary Report. 2014.

19. ICHOM. International Consortium for Health Outcomes Measurement [Internet]. Available from: https://www.ichom.org/why-measure-outcomes/

20. Orrego C, Ballester M, Pacheco-Barrios K, Camus E, Heymans M, Groene O, et al. Development and external validation of a comprehensive Taxonomy of Self-Management Interventions in chronic conditions: the COMPAR-EU taxonomy. In 2019. Available from: https://self-management.eu/wp-content/uploads/2019/11/7863_Taxonomy_GIN_Poster.pdf

21. Niño de Guzmán E, Martínez-García L, González AI, Heijmans M, Huaringa J, Immonen K, et al. The perspectives of patients and their caregivers on self-management interventions for chronic conditions: a protocol for a mixed-methods overview. F1000Research. 2020 Feb 18;9:120.

22. Selva A, Solà I, Zhang Y, Pardo-Hernandez H, Haynes RB, Martínez García L, et al. Development and use of a content search strategy for retrieving studies on patients' views and preferences. Health Qual Life Outcomes. 2017 Dec 30;15(1):126.

23. European Patients Forum [Internet]. 2020. Available from: https://www.eu-patient.eu

24. McLean S, Nurmatov U, Liu JL, Pagliari C, Car J, Sheikh A. Telehealthcare for chronic obstructive pulmonary disease. Cochrane Database Syst Rev. 2011 Jul 6;

25. Vieira DSR, Maltais F, Bourbeau J. Home-based pulmonary rehabilitation in chronic obstructive pulmonary disease patients. Curr Opin Pulm Med. 2010 Mar;16(2):134–43.

26. Kruis AL, Smidt N, Assendelft WJJ, Gussekloo J, Boland MRS, Rutten-van Mölken M, et al. Integrated disease management interventions for patients with chronic

obstructive pulmonary disease. Cochrane database Syst Rev. 2013 Oct 10;(10):CD009437.

27. Bryant J, McDonald VM, Boyes A, Sanson-Fisher R, Paul C, Melville J. Improving medication adherence in chronic obstructive pulmonary disease: a systematic review. Respir Res. 2013 Oct 20;14:109.

28. Health Quality Ontario. In-home care for optimizing chronic disease management in the community: an evidence-based analysis. Ont Health Technol Assess Ser. 2013;13(5):1–65.

29. Quiñones AR, Richardson J, Freeman M, Fu R, O'Neil ME, Motu'apuaka M, et al. Educational group visits for the management of chronic health conditions: a systematic review. Patient Educ Couns. 2014 Apr;95(1):3–29.

30. Prieto-Centurion V, Markos MA, Ramey NI, Gussin HA, Nyenhuis SM, Joo MJ, et al. Interventions to reduce rehospitalizations after chronic obstructive pulmonary disease exacerbations. A systematic review. Ann Am Thorac Soc. 2014 Mar;11(3):417–24.

31. Li G, Yuan H, Zhang W. Effects of Tai Chi on health related quality of life in patients with chronic conditions: a systematic review of randomized controlled trials. Complement Ther Med. 2014 Aug;22(4):743–55.

32. Zwerink M, Brusse-Keizer M, van der Valk PDLPM, Zielhuis GA, Monninkhof EM, van der Palen J, et al. Self management for patients with chronic obstructive pulmonary disease. Cochrane database Syst Rev. 2014 Mar 19;(3):CD002990.

33. Harrison SL, Janaudis-Ferreira T, Brooks D, Desveaux L, Goldstein RS. Self-management following an acute exacerbation of COPD: a systematic review. Chest. 2015 Mar;147(3):646–61.

34. McCarthy B, Casey D, Devane D, Murphy K, Murphy E, Lacasse Y. Pulmonary rehabilitation for chronic obstructive pulmonary disease. Cochrane database Syst Rev. 2015 Feb 23;(2):CD003793.

35. Majothi S, Jolly K, Heneghan NR, Price MJ, Riley RD, Turner AM, et al. Supported self-management for patients with COPD who have recently been discharged from hospital: a systematic review and meta-analysis. Int J Chron Obstruct Pulmon Dis. 2015;10:853–67.

36. Polisena J, Tran K, Cimon K, Hutton B, McGill S, Palmer K, et al. Home telehealth for chronic obstructive pulmonary disease: a systematic review and meta-analysis. J Telemed Telecare. 2010;16(3):120–7.

37. Flodgren G, Rachas A, Farmer AJ, Inzitari M, Shepperd S. Interactive telemedicine: effects on professional practice and health care outcomes. Cochrane database Syst Rev. 2015 Sep 7;(9):CD002098.

38. Jolly K, Majothi S, Sitch AJ, Heneghan NR, Riley RD, Moore DJ, et al. Self-management of health care behaviors for COPD: a systematic review and meta-analysis. Int J Chron Obstruct Pulmon Dis. 2016;11:305–26.

39. Walters JA, Turnock AC, Walters EH, Wood-Baker R. Action plans with limited patient education only for exacerbations of chronic obstructive pulmonary disease. Cochrane database Syst Rev. 2010 May 12;(5):CD005074.

40. Gosselink R, De Vos J, van den Heuvel SP, Segers J, Decramer M, Kwakkel G. Impact of inspiratory muscle training in patients with COPD: what is the evidence? Eur Respir J. 2011 Feb;37(2):416–25.

41. Bryant J, Bonevski B, Paul C, McElduff P, Attia J. A systematic review and meta-analysis of the effectiveness of behavioural smoking cessation interventions in

selected disadvantaged groups. Addiction. 2011 Sep;106(9):1568–85.
42. Cindy Ng LW, Mackney J, Jenkins S, Hill K. Does exercise training change physical activity in people with COPD? A systematic review and meta-analysis. Chron Respir Dis. 2012 Feb;9(1):17–26.
43. Hamilton FL, Greaves F, Majeed A, Millett C. Effectiveness of providing financial incentives to healthcare professionals for smoking cessation activities: systematic review. Tob Control [Internet]. 2013 Jan;22(1):3–8. Available from: http://www.ncbi.nlm.nih.gov/pubmed/22123941
44. Collins PF, Stratton RJ, Elia M. Nutritional support in chronic obstructive pulmonary disease: a systematic review and meta-analysis. Am J Clin Nutr. 2012 Jun;95(6):1385–95.
45. Tan J-Y, Chen J-X, Liu X-L, Zhang Q, Zhang M, Mei L-J, et al. A Meta-Analysis on the Impact of Disease-Specific Education Programs on Health Outcomes for Patients with Chronic Obstructive Pulmonary Disease. Geriatr Nurs (Minneap). 2012 Jul;33(4):280–96.
46. Cazzola M, MacNee W, Martinez FJ, Rabe KF, Franciosi LG, Barnes PJ, et al. Outcomes for COPD pharmacological trials: from lung function to biomarkers. Eur Respir J. 2008 Feb 1;31(2):416–69.
47. Jadad A, Rizo C, Cubillos P, Ståhl E. Measuring symptom response to pharmacological interventions in patients with COPD: a review of instruments used in clinical trials. Curr Med Res Opin. 2004 Dec 24;20(12):1993–2005.
48. Einarson T, Bereza B, Nielsen A, Hemels M, Valgardsson S. Patient preferences in severe COPD and asthma: a comprehensive literature review. Int J Chron Obstruct Pulmon Dis. 2015 Apr;739.

49. Bulcun E, Ekici A, Ekici M. Assessment of patients' preferences regarding the characteristics associated with the treatment of chronic obstructive pulmonary disease. Int J Chron Obstruct Pulmon Dis. 2014 Apr;9:363–8.

50. Glaab T, Vogelmeier C, Buhl R. Outcome measures in chronic obstructive pulmonary disease (COPD): strengths and limitations. Respir Res. 2010 Dec 17;11(1):79.

51. Bjermer L. The Importance of Continuity in Inhaler Device Choice for Asthma and Chronic Obstructive Pulmonary Disease. Respiration. 2014;88(4):346–52.

52. Brundisini F, Giacomini M, DeJean D, Vanstone M, Winsor S, Smith A. Chronic disease patients' experiences with accessing health care in rural and remote areas: A systematic review and qualitative meta-synthesis. Ont Health Technol Assess Ser. 2013;13(15):1–33.

53. Brunton L, Bower P, Sanders C. The Contradictions of Telehealth User Experience in Chronic Obstructive Pulmonary Disease (COPD): A Qualitative Meta-Synthesis. Soyer HP, editor. PLoS One [Internet]. 2015 Oct 14;10(10):e0139561. Available from: https://dx.plos.org/10.1371/journal.pone.0139561

54. Lindenmeyer A, Greenfield SM, Greenfield C, Jolly K. How Do People With COPD Value Different Activities? An Adapted Meta-Ethnography of Qualitative Research. Qual Health Res. 2017 Jan 9;27(1):37–50.

55. Mathar H, Fastholm P, Hansen IR, Larsen NS. Why Do Patients with COPD Decline Rehabilitation. Scand J Caring Sci. 2016 Sep;30(3):432–41.

56. Russell S, Ogunbayo OJ, Newham JJ, Heslop-Marshall K, Netts P, Hanratty B, et al. Qualitative systematic review of barriers and facilitators to self-management

of chronic obstructive pulmonary disease: views of patients and healthcare professionals. npj Prim Care Respir Med. 2018 Dec 17;28(1):2.

57. Sohanpal R, Steed L, Mars T, Taylor SJC. Understanding patient participation behaviour in studies of COPD support programmes such as pulmonary rehabilitation and self-management: a qualitative synthesis with application of theory. npj Prim Care Respir Med. 2015 Dec 17;25(1):15054.

58. Gorst SL, Armitage CJ, Brownsell S, Hawley MS. Home Telehealth Uptake and Continued Use Among Heart Failure and Chronic Obstructive Pulmonary Disease Patients: a Systematic Review. Ann Behav Med. 2014 Dec 25;48(3):323–36.

59. Habraken JM, Willems DL, de Kort SJ, Bindels PJE. Health care needs in end-stage COPD: A structured literature review. Patient Educ Couns. 2007 Oct;68(2):121–30.

60. Clari M, Ivziku D, Casciaro R, Matarese M. The Unmet Needs of People with Chronic Obstructive Pulmonary Disease: A Systematic Review of Qualitative Findings. COPD J Chronic Obstr Pulm Dis. 2018 Jan 2;15(1):79–88.

61. Clari M, Matarese M, Ivziku D, De Marinis MG. Self-Care of People with Chronic Obstructive Pulmonary Disease: A Meta-Synthesis. Patient - Patient-Centered Outcomes Res. 2017 Aug 14;10(4):407–27.

62. de Sousa Pinto JM, Martín-Nogueras AM, Morano MTAP, Macêdo TEPM, Arenillas JIC, Troosters T. Chronic obstructive pulmonary disease patients' experience with pulmonary rehabilitation: A systematic review of qualitative research. Chron Respir Dis. 2013 Aug 29;10(3):141–57.

63. Disler RT, Green A, Luckett T, Newton PJ, Inglis S, Currow DC, et al. Experience of Advanced Chronic Obstructive Pulmonary Disease: Metasynthesis of Qualitative

Research. J Pain Symptom Manage. 2014 Dec;48(6):1182–99.

64. Giacomini M, DeJean D, Simeonov D, Smith A. Experiences of living and dying with COPD: A systematic review and synthesis of the qualitative empirical literature. Ont Health Technol Assess Ser. 2012;12(13):1–47.

65. Gysels M, Bausewein C, Higginson IJ. Experiences of breathlessness: A systematic review of the qualitative literature. Palliat Support Care. 2007 Sep 24;5(3):281–302.

66. Harrison C, Britt H, Miller G, Henderson J. Examining different measures of multimorbidity, using a large prospective cross-sectional study in Australian general practice. BMJ Open [Internet]. 2014;4(7):e004694. Available from: http://www.pubmedcentral.nih.gov/articlerender.fcgi?artid=4120329&tool=pmcentrez&rendertype=abstract

67. Kirkpatrick P, Wilson E, Wimpenny P. Support for Older People with COPD in Community Settings: A Systematic Review of Qualitative Research. JBI Database Syst Rev Implement Reports. 2012;10(57):3649–763.

68. Dretzke J, Blissett D, Dave C, Mukherjee R, Price M, Bayliss S, et al. The cost-effectiveness of domiciliary non-invasive ventilation in patients with end-stage chronic obstructive pulmonary disease: a systematic review and economic evaluation. Health Technol Assess (Rockv). 2015 Oct;19(81):1–246.

69. Jordan RE, Majothi S, Heneghan NR, Blissett DB, Riley RD, Sitch AJ, et al. Supported self-management for patients with moderate to severe chronic obstructive pulmonary disease (COPD): an evidence synthesis and economic analysis. Health Technol Assess (Rockv). 2015 May;19(36):1–516.

70. Moayeri F, Hsueh Y-S (Arthur), Clarke P, Dunt D. Do Model-Based Studies in

Chronic Obstructive Pulmonary Disease Measure Correct Values of Utility? A Meta-Analysis. Value Heal. 2016 Jun;19(4):363–73.

71. Rose S, Paul C, Boyes A, Kelly B, Roach D. Stigma-related experiences in non-communicable respiratory diseases: A systematic review. Chron Respir Dis. 2017 Aug 23;14(3):199–216.

72. Zhang Y, Morgan RL, Alonso-Coello P, Wiercioch W, Bała MM, Jaeschke RR, et al. A systematic review of how patients value COPD outcomes. Eur Respir J. 2018 Jul;52(1):1800222.

73. Cox NS, Oliveira CC, Lahham A, Holland AE. Pulmonary rehabilitation referral and participation are commonly influenced by environment, knowledge, and beliefs about consequences: a systematic review using the Theoretical Domains Framework. J Physiother. 2017 Apr;63(2):84–93.

74. Kaptein AA, Scharloo M, Fischer MJ, Snoei L, Cameron LD, Sont JK, et al. Illness Perceptions and COPD: An Emerging Field for COPD Patient Management. J Asthma. 2008 Jan 2;45(8):625–9.

75. Keating A, Lee A, Holland AE. What prevents people with chronic obstructive pulmonary disease from attending pulmonary rehabilitation? A systematic review. Chron Respir Dis. 2011 May;8(2):89–99.

76. Sørensen SS, Weinreich UM, Ehlers L. Rationale and development of a patient-tailored complex intervention of case management for patients suffering from chronic obstructive pulmonary disease. Home Health Care Serv Q. 2017 Oct 2;36(3–4):178–95.

77. Rosa F, Bagnasco A, Aleo G, Kendall S, Sasso L. Resilience as a concept for understanding family caregiving of adults with Chronic Obstructive Pulmonary

Disease (COPD): an integrative review. Nurs Open. 2017 Apr;4(2):61–75.

78. Kirkham JJ, Gorst S, Altman DG, Blazeby JM, Clarke M, Devane D, et al. Core Outcome Set–STAndards for Reporting: The COS-STAR Statement. PLOS Med. 2016 Oct 18;13(10):e1002148.

79. Williamson PR, Altman DG, Blazeby JM, Clarke M, Devane D, Gargon E, et al. Developing core outcome sets for clinical trials: issues to consider. Trials. 2012 Dec 6;13(1):132.

80. Jones JE, Jones LL, Keeley TJH, Calvert MJ, Mathers J. A review of patient and carer participation and the use of qualitative research in the development of core outcome sets. Young B, editor. PLoS One. 2017 Mar 16;12(3):e0172937.

81. Spargo M, Ryan C, Downey D, Hughes C. Development of a core outcome set for trials investigating the long-term management of bronchiectasis. Chron Respir Dis. 2019 Jan 3;16:147997231880416.

82. Verburg AC, van Dulmen SA, Kiers H, Ypinga JH, Nijhuis-van der Sanden MW, van der Wees PJ. Development of a Standard Set of Outcome Domains and Proposed Measures for Chronic Obstructive Pulmonary Disease in Primary Care Physical Therapy Practice in the Netherlands: a Modified RAND/UCLA Appropriateness Method. Int J Chron Obstruct Pulmon Dis. 2019 Nov;Volume 14:2649–61.

83. Jones A V, Evans RA, Man WD-C, Bolton CE, Breen S, Doherty PJ, et al. Outcome measures in a combined exercise rehabilitation programme for adults with COPD and chronic heart failure: A preliminary stakeholder consensus event. Chron Respir Dis. 2019 Jan 1;16:147997311986795.

84. Harrison SL, Apps L, Singh SJ, Steiner MC, Morgan MD, Robertson N. ‘Consumed

by breathing' – a critical interpretive meta-synthesis of the qualitative literature. Chronic Illn. 2014 Mar 13;10(1):31–49.

85. Brunton L, Bower P, Sanders C. The Contradictions of Telehealth User Experience in Chronic Obstructive Pulmonary Disease (COPD): A Qualitative Meta-Synthesis. Soyer HP, editor. PLoS One. 2015 Oct 14;10(10):e0139561.

86. Sørensen SS, Weinreich UM, Ehlers L. Rationale and development of a patient-tailored complex intervention of case management for patients suffering from chronic obstructive pulmonary disease. Home Health Care Serv Q [Internet]. 2017 Oct 2;36(3–4):178–95. Available from: https://www.tandfonline.com/doi/full/10.1080/01621424.2017.1393481

87. Einarson TR, Bereza BG, Nielsen TA, Hemels MEH. Utilities for asthma and COPD according to category of severity: a comprehensive literature review. J Med Econ. 2015 Jul 3;18(7):550–63.

Table 1. Categories of outcomes by level of agreement

Group	Votes		Interpretation
Group 1a	≥ 70% voted 8-9	≤ 15% voted 1-3	High agreement on high importance. Suggestion to include on Core Outcome Set
Group 1b	≥ 70% voted 7	≤ 15% voted 1-3	High agreement on high importance. Suggestion to include on Core Outcome Set
Group 2	Intermediate results		Inclusion or exclusion on Core Outcome Set to be decided in consensus workshop
Group 3	≤ 15% voted 8-9	≥ 70% voted 1-3	High agreement on moderate or low importance. Suggestion to exclude from Core Outcome Set

Table 2. List of COPD outcomes and classification

Subdomain	Outcome
Basic empowerment components	1. Patient activation
	2. Self-efficacy
	3. Knowledge
	4. Health literacy
	5. Caregiver knowledge
	6. Caregiver self-efficacy
Level of adherence to expected self-management behaviors	7. Taking medication or other treatment as advised (adherence)
	8. Self-monitoring
	9. Diet habits
	10. Diet habits (adherence to diet)
	11. Physical activity
	12. Smoking cessation
	13. Smoking
Clinical outcomes	14. Body weight
	15. Malnutrition
	16. Tiredness (fatigue)
	17. Interrupted
	18. Sleep problems sleep (disturbed sleep)
	19. Sleep quality
	20. Sleepiness
	21. Chest tightness or discomfort
	22. COPD symptoms (short term)
	23. COPD symptoms (long term)
	24. Breathlessness (Dyspnea)
	25. Exacerbation
	26. Lung function (FEV1, FVC)
	27. Lung function
	28. Lung function (LTOT)
	29. Lung function / CPAP
	30. Muscle strength
	31. Effort test / Exercise capacity
	32. Complications
	33. Treatment side effects (adverse effects)
	34. Mortality
Patient and informal caregivers' quality of life	35. Usual activities
	36. Mobility
	37. Work
	38. Physical activities
	39. Sex life
	40. Normality

	41. Pain or discomfort
	42. Treatment burden
	43. Medication burden
	44. Positive attitude
	45. Depression
	46. Anxiety
	47. Stress
	48. Coping
	49. Hostility
	50. Happiness
	51. Participation in social activities
	52. Self-esteem
	53. Family relationships
	54. Friends
	55. Social activities
	56. Caregiver quality of life
	57. Caregiver burden
	58. Caregiver anxiety and / or depression
Perception of and / or satisfaction with care	59. Satisfaction with / perception of care
	60. Participation and decision-making
	61. Patient-health care provider relation
	62. Communication with health care professionals
	63. Extent to which the health care professional gives enough time to listen to the patient
	64. The patient feels s/he has enough information
Healthcare use	65. Number of primary care or outpatient (ambulatory) visits
	66. Number of nurse visits
	67. Number of virtual visits or contacts with healthcare providers
	68. Number of visits to specialist doctors
	69. Number of home care visits
	70. Number of visits with other healthcare professionals
	71. (Number of) emergency department visits (hospital)
	72. Number of hospital admissions

	73. The length of time spent in hospital (length of hospital stays)
	74. Number of re-hospitalizations unexpected return to hospital
Cost	75. Impact of healthcare costs for the healthcare system
	76. Cost of hospitalizations for the healthcare system
	77. Cost savings for the healthcare system
	78. Direct medical costs for patient
	79. Value for money of the self-management intervention

Table 3. Main themes related to COPD outcomes according to the subdomains from COMPAR-EU taxonomy

Subdomains from the COMPAR-EU taxonomy	Main themes for COPD	References
Empowerment components	Health knowledge (52,56,57,60,63,64)	(52,56,57,60,63,64)
	Help/health-seeking behavior	(52,60,64,65,84)
	Technological (digital) literacy	(58)
Adherence to expected self-management behaviors	Adherence to treatment	(65)
	Self-care ability	(54,74,84,85)
	Smoking behavior	(56,64,65,67,71)
	Perceived benefit (importance) of the intervention	(55–58,62,67,73,75,86)
Clinical-related outcomes	Adverse events	(72)
	Mortality	(56)
	Progression of the disease	(63–65,68–70,72,84,87)
Quality of life of patients and caregivers	Informal caregiver's' burden	(64,65,77)
	Physical functioning	(56,60,62–64,75,76)
	Psychological distress	(56,58,61,63,65,71,74,75,85)
	Social support	(52,55,76,84,85,56,57,61–63,67,73,75)
Perceptions and/or satisfaction with care	Individualized care	(65)
	Patient-provider interaction	(52,63–65,84)
	Perceived quality of care	(58,85)
	Usability	(58,67)
Healthcare use	Access to healthcare	(52,60,64)
	Visits or contacts with healthcare professionals	(58,67,85)
Costs	Cost for patients (out of pocket)	(72)

COPD = Chronic Obstructive Pulmonary Disease

Table 4. COMPAR-EU COS for COPD

Outcome (COS)	Definition
Knowledge (supplementary)	Relates to knowledge about COPD in general and COPD self-management, or the way care for COPD is organized and this both for patients and their social network.
Caregiver knowledge and competence	That the caregiver has competences and knowledge of the disease and its management.
Self-efficacy	A person's belief that s/he is capable of doing something, often related to a specific goal s/he wants to achieve; feeling of confidence and of being in control.
Patient activation	The knowledge, skills and confidence a person has on managing their own health and healthcare, including a feeling of being responsible for taking care of their own health.
Self-monitoring	The extent to which a patient (regularly) monitors themselves as agreed with her/his healthcare professionals, for example her/his symptoms or weight.
Taking medication or other treatment as advised (adherence) and adherence to regular visits	The extent to which a patient follows the prescribed treatment, such as taking medication as advised and following life-style advice, and extent of attending scheduled visits.
Smoking cessation	Stopping smoking (and/or smoking less).
COPD symptoms (short term)	Extent of Symptoms relief (in the short-term, including cough; breathlessness, among others).
Physical activity - muscle strength	Referral/participation in a Pulmonary Rehabilitation program: Physical activity, Physical activities, Muscle strength linked with exercise capacity plus an overall support.
Sleep quality	Sleep quality contains interrupted sleep, sleep problems, sleep quality (as overall) and sleepiness.
Exacerbation	Increased breathlessness, mucus/phlegm/sputum production, and change in color of sputum and Feeling out of breath.
Caregiver quality of life (including burden)	Caregiver quality of life and the burden that he/she feels from the caregiver's tasks.
Activities of daily living: including sex life, social activities and work (usual activities)	Being able to do usual activities, such as personal hygiene, housework, sex, managing finances, social activities and work.
Coping with the disease, including depression and anxiety	How well a person feels able to cope/manage with stress or other difficulties caused by the disease, including depression and anxiety.
Participation and decision making	Feeling able to participate actively in her/his own care (as much as s/he wishes).
Number of emergency room	Number of visits to emergency department visits and hospital admissions.

visits and admissions	
Cost effectiveness and resources use	It includes value for money of the self-management intervention and the use of resources.

Figure 1. Overall study design of Core Outcome Set for COPD

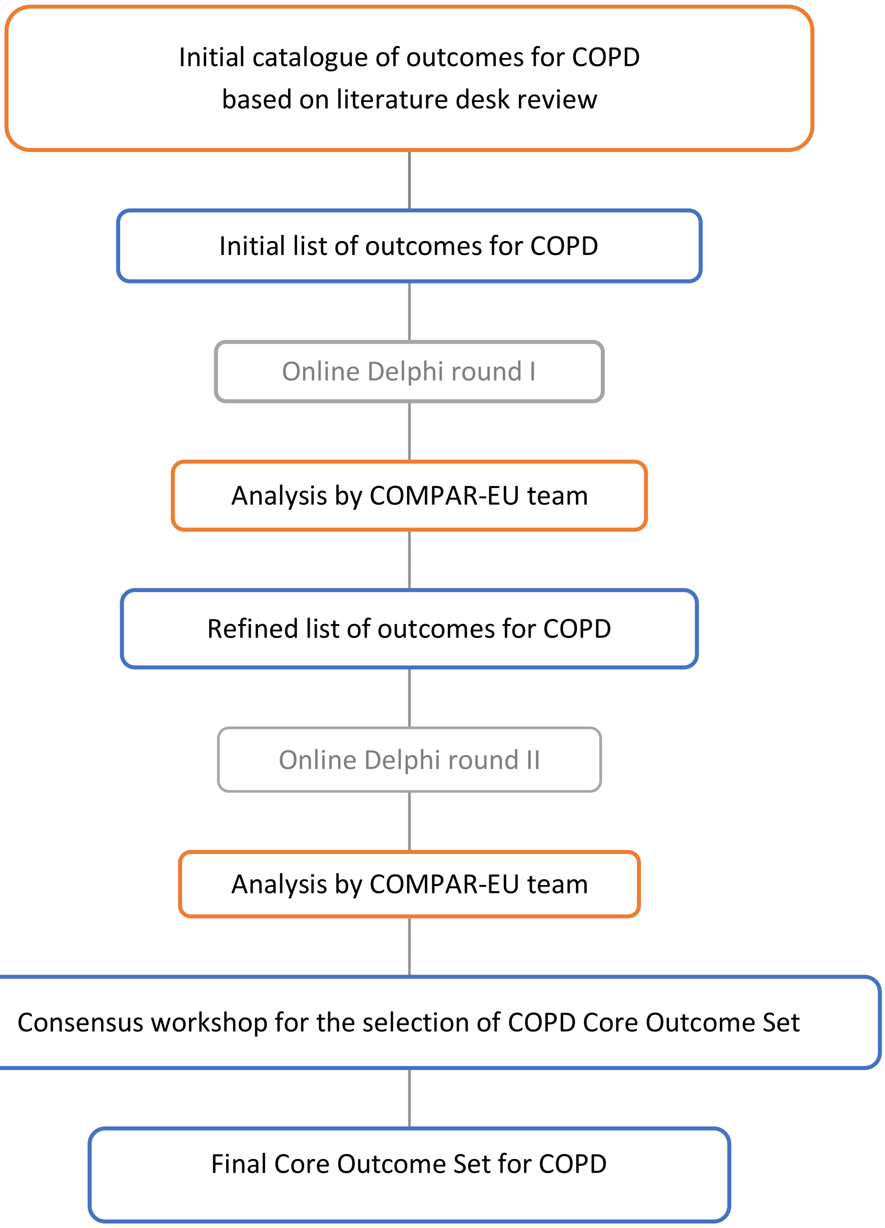

COPD: Chronic Obstructive Pulmonary Disease

Figure 2. Consensus workshop decision diagram

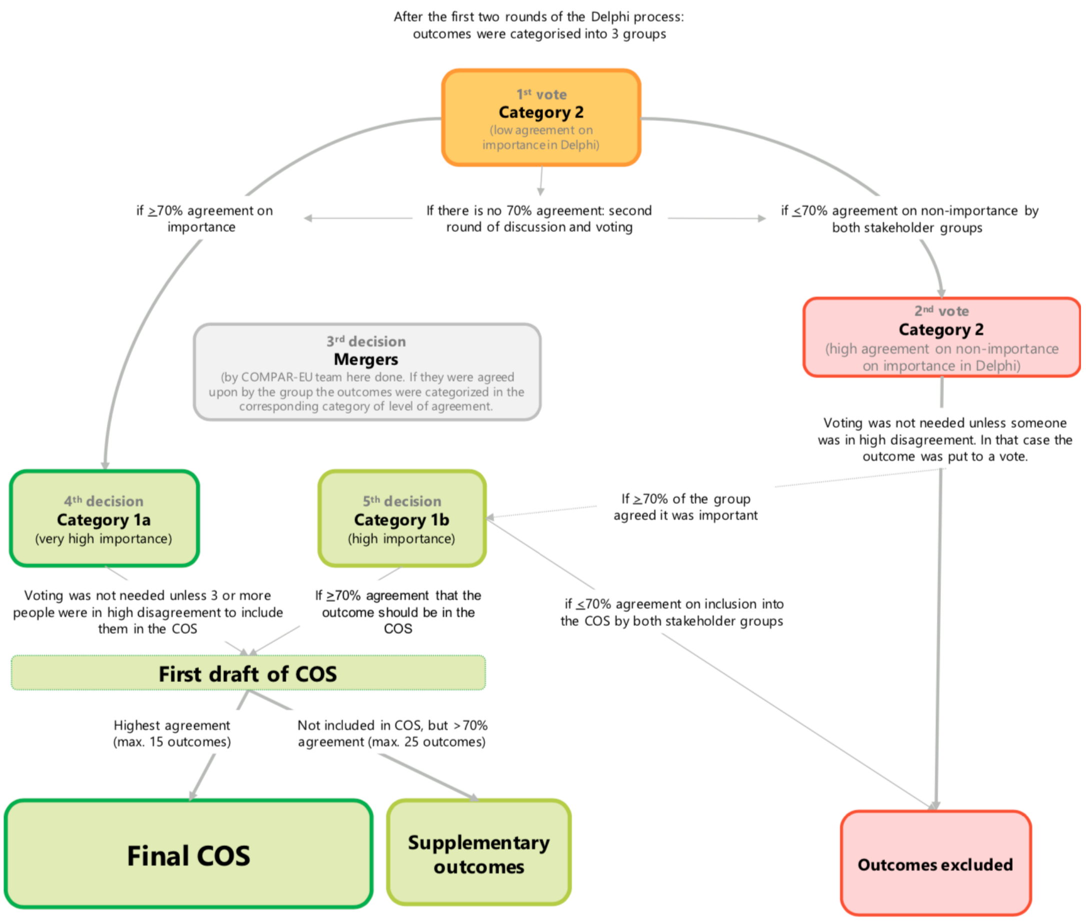

Click here to access/download;Figure;Figure 3.pdf

Figure 3. Mapping of themes per COMPAR-EU taxonomy subdomain

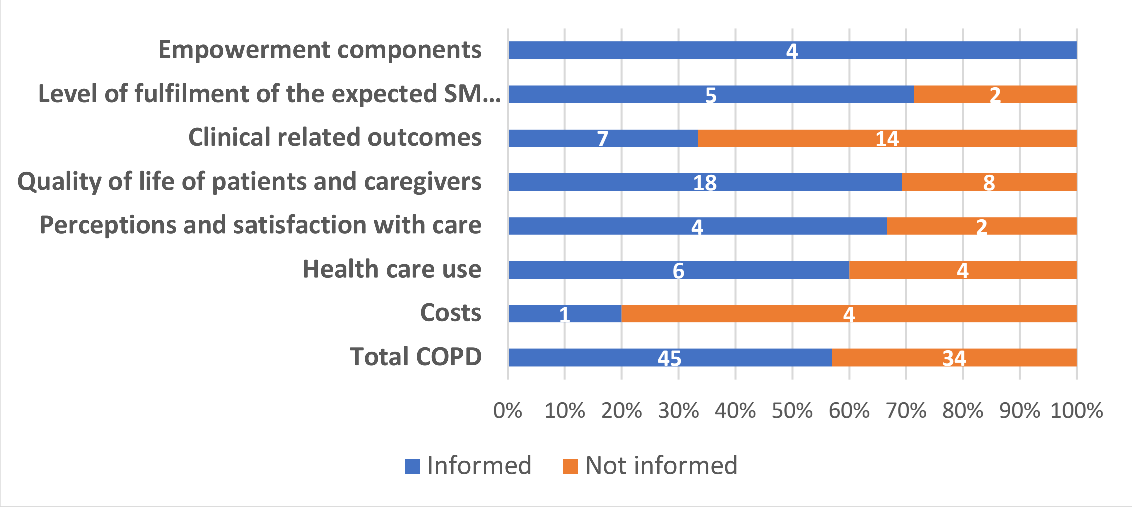

Number of outcomes informed or not informed by the thematic synthesis of the scoping review.

Supplementary file 1. Infographic COPD

Click here to access/download
Supporting Information
Supplementary file 1. Infographic COPD.pdf

Supplementary file 2. Delphi online survey participants characteristics

Click here to access/download
Supporting Information
Supplementary file 2_20200702.pdf

Click here to access/download
Supporting Information
Supplementary file 3_20200702.pdf

Supplementary file 4

Click here to access/download
Supporting Information
Supplementary file 4_20200702.pdf
